# Exploring the experiences, challenges, and coping strategies of caregivers of women with Ovarian Cancer: A scoping review

**DOI:** 10.1371/journal.pone.0345325

**Published:** 2026-04-30

**Authors:** Gabrielle C. Smith, Matthew R. Langiano, Junhee Baek, Esme Fuller-Thomson

**Affiliations:** 1 Factor-Inwentash Faculty of Social Work, University of Toronto, Toronto, Ontario, Canada; 2 Department of Supportive Care, Princess Margaret Cancer Centre, University Health Network, Toronto, Ontario, Canada; 3 Institute for Life Course and Aging, University of Toronto, Toronto, Ontario, Canada; The Hong Kong Polytechnic University, HONG KONG

## Abstract

**Background:**

Ovarian cancer (OC) is among the most lethal gynecological malignancies and a leading cause of cancer-related deaths in women. Significant attention has been given to the experiences of OC patients; however, the experiences of their family caregivers have been largely overlooked.

**Objectives:**

The objectives of this scoping review is to characterize the scope, nature and focus of the existing literature describing the experiences of caregivers’ of individuals with OC during the caregiving process and after the care recipient’s death, and to identify gaps in the literature.

**Methods:**

This scoping review was guided by Arksey and O’Malley’s (2005) framework. Comprehensive searches were conducted in MEDLINE, EMBASE, CINAHL, PsycINFO, and Social Work Abstracts to identify literature on caregivers of individuals living with OC. A total of 739 titles and abstracts were screened by two reviewers, 123 full-text articles were assessed, and 32 met inclusion criteria for data extraction. Themes were extracted after the reviewers carefully read through the selected articles.

**Results:**

Main themes included (1) the well-being and mental health of caregivers, with two sub-themes (a) emotional well-being and mental health, (b) grief, bereavement and post-traumatic stress disorder; (2) challenges facing caregivers, with seven sub-themes (a) escalating patients needs and patient experiences, (b) interactions with doctors and medical system, (c) social isolation, (d) family responsibilities, (e) work stressors and financial burdens, (f) health behaviours and inadequate self-care, and (g) intrapersonal stressors; and (3) positive coping strategies with sub-themes (a) spirituality and (b) social support.

**Conclusion:**

This scoping review highlights an urgent need for tailored support for OC caregivers and may provide valuable insights for clinical practice in the field of caregiver wellbeing. Future research and greater awareness of the lived experience of OC caregivers may inform policymakers to develop policy initiatives promoting more family-centered care.

## Introduction

Ovarian cancer (OC) is a highly lethal gynecological malignancy, with over 313,000 diagnoses and 207,000 deaths reported worldwide in 2020 [1]. Late-stage diagnosis is particularly prevalent, with approximately 75% of cases identified at stage III or IV due to the absence of effective early screening tools [2]. These factors contribute to a five-year survival rate of less than 50% for most patients diagnosed at advanced stages [3]. Recurrence is another prominent and well-documented challenge, with approximately 70% of OC patients experiencing recurrence following initial treatment [4]. Despite comprising only 1.3% of new cancer cases annually, OC is the fifth-leading cause of cancer deaths among women in the United States [2]. These factors emphasize the challenges posed by OC’s aggressive nature and the need for improved early detection strategies [3].

Caregivers play a significant, yet frequently overlooked, role in supporting individuals living with cancer. Acting as the primary support system, caregivers often manage medical appointments, provide physical care and offer emotional reassurance, making their contributions indispensable to the patient. This demanding role often extends beyond the caregiving dyad, affecting caregivers’ other relationships, professional responsibilities, and overall quality of life [5]. Furthermore, extensive caregiving responsibilities have been associated with negative outcomes, such as physical exhaustion and psychological distress [6,7].

The burden of caregiving often extends beyond emotional distress, with caregivers facing heightened risks of depression, anxiety, and post-traumatic stress disorder (PTSD) [8]. In fact, research indicates that caregivers of individuals with cancer experience notably high rates of grief-related conditions, including Prolonged Grief Disorder and Persistent Complex Bereavement Disorder [9], as well as anticipatory grief that emerges prior to the patient’s death [10].

The caregivers’ physical health may also suffer; Caregivers report high rates of chronic conditions like hypertension and sleep disturbances. Caregivers often neglect their own medical needs because they are so busy with attending to their caregiving responsibilities [11]. Moreover, there is limited evidence on mental health interventions specifically tailored to caregivers [12]. Systemic gaps in resources, such as counseling and respite care, highlight the urgent need for targeted support to enhance caregiver well-being and patient outcomes [13]. Financial strain is also a common challenge among caregivers, particularly for those from lower socioeconomic backgrounds, who face greater monetary stress and limited access to resources [14,15].

For caregivers of OC patients, these challenges are particularly acute. The unpredictable nature of OC, characterized by prolonged treatment trajectories, intensive symptom management, and frequent recurrence after a period of remission significantly exacerbates caregivers’ stress [16]. Research suggests that the caregiver stress level, mental health, and quality of life are directly linked to the emotional and physical health of adult cancer patients, making the caregiver-patient relationship a critical element of cancer care [13]. Emerging interventions, including psychoeducation, mindfulness-based stress reduction, and peer support groups, have shown promise in reducing caregiver burden; however, their application and effectiveness in OC caregiving still needs to be explored [12]. While caregiving in other cancer contexts has received substantial attention, the unique experiences of OC caregivers remain underexplored.

A previous scoping review by Petricone-Westwood and Lebel [17] was the first to map the literature on caregivers of individuals with ovarian cancer, identifying 19 relevant studies published prior to November 2014. Their review provided an important foundation for understanding the psychological distress, unmet needs, and quality-of-life challenges experienced by caregivers across the disease trajectory. However, given the substantial growth in caregiving and psycho-oncology research over the past decade, particularly concerning bereavement experiences, gender differences, and coping mechanisms, an updated synthesis is warranted. The current scoping review therefore serves as an updated synthesis of the literature, expanding on the earlier work by incorporating studies published between 2014 and 2024, employing a broader database search strategy, and revisiting those publications published prior to 2014 identified in Petricone-Westwood and Lebel’s review to provide a more integrated synthesis.

Despite growing research on cancer caregiving, significant gaps remain in understanding the unique stressors, coping strategies, and support needs of caregivers to OC patients [17]. Coping in the caregiving context is especially significant as adaptive approaches to coping, such as acceptance, positive reframing, and help-seeking, are associated with better psychological adjustment; while avoidance and denial are linked to greater distress and burnout [18]. This highlights the importance of targeted interventions that support the needs of this specific demographic and their contextual circumstances [19].

Addressing these gaps is essential for developing interventions that enhance OC caregivers’ well-being and potentially improving patient outcomes. This scoping review maps the emerging themes in the existing literature on the experiences of OC caregivers and identify gaps in the research. To achieve these goals, this scoping review’s purpose was to document the lived experiences of caregivers of individuals with OC during the caregiving process and after the care recipient’s death. We were particularly interested in the mental and physical health of caregivers; factors associated with positive and/or negative caregiver well-being, and coping strategies they adopted. A final objective was to identify gaps in the existing literature and highlight areas for future research to better understand and address the needs of OC caregivers.

## Methods

This review was conducted following the six-step methodological framework for scoping review by Arksey and O’Malley [20] to analyze and summarize the available literature on the experiences of caregivers of individuals living with OC. The review was also reported in accordance with the PRISMA-ScR (Preferred Reporting Items for Systematic reviews and Meta-Analyses extension for Scoping Reviews) checklist [21] to ensure transparency and completeness. We did not register a protocol for this scoping review and no publicly accessible protocol exists.

### Step 1: Identifying the research question

This review was guided by the following question: What is known about the experiences of caregivers of individuals with ovarian cancer (OC) during caregiving and following the care recipient’s death?

### Step 2: Identifying relevant studies

An electronic search of the literature was conducted on October 22, 2024. Five databases were selected due to their focus on medical and allied health sciences: PsycINFO (1946-), Medline (1946-), Embase (1946-), Social Work Abstracts (1968-), and CINAHL (1983-). The reference list of all articles which were included in our study, were manually searched for additional relevant articles that may not have been identified through the database searches. In order to ensure that the current review was comprehensive, we conducted forward citation searching on all articles which met our inclusion criteria. Each article was entered into Google Scholar and we conducted a titles and abstracts screening of all sources citing the original article. Through this process we identified 3 additional publications that met the inclusion and exclusion criteria and were therefore included in the scoping review.

A collection of search terms was curated based on the following two concepts: OC, caregivers. To generate the search terms used, we first conducted a web search for synonyms of ovary, cancer and OC, and then we conducted a search of subject heading terms/MEsH terms from the five databases we consulted. Please see S3 Table for all search terms used for this scoping review.

### Step 3: Study selection

**Fig 1:**
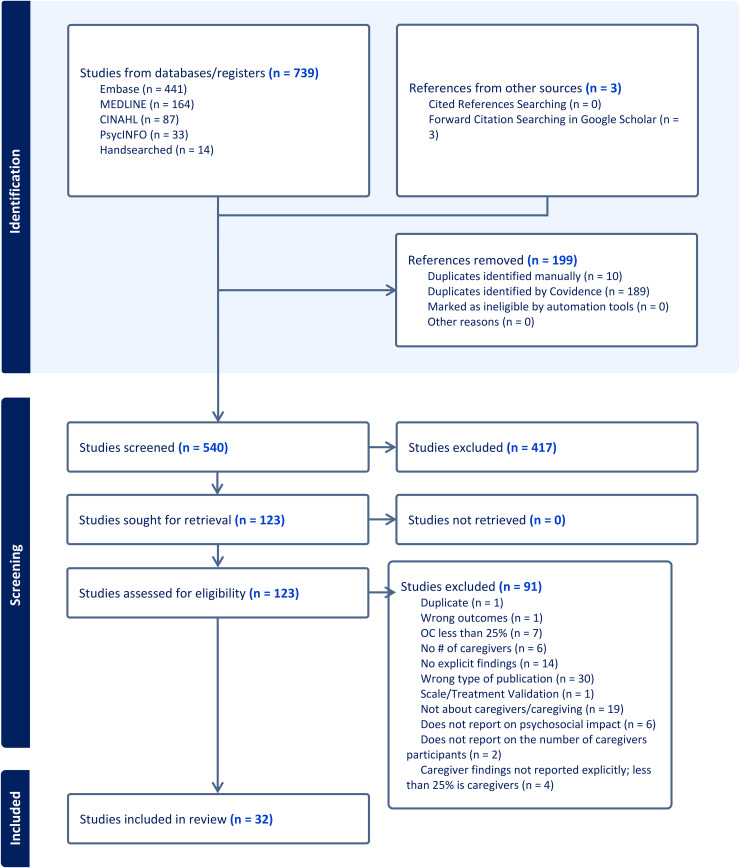
PRISMA ScR flow chart.

Once the search was completed, the screening process was conducted using Covidence, a web-based software used for scoping and systematic reviews [22,23]. The PRISMA flow chart (see Fig 1), created using Covidence [24], illustrates the detailed process of identification and screening for this scoping review. The completed PRISMA-ScR checklist is provided in S1 Table.

The present scoping review included peer‑reviewed, English‑language publications and book chapters using narrative, case study, quantitative, qualitative, or review designs. All sources needed to examine caregivers supporting individuals with ovarian cancer (OC). Because our aim was to map the breadth and scope of existing knowledge rather than limit the review to specific empirical methods, we also included peer‑reviewed books, narrative reports, and reviews that offered original qualitative or contextual insights into caregivers’ experiences.

These sources contributed conceptual data (e.g., lived-experience accounts or model development) not captured in quantitative studies. However, we recognize that including reviews may introduce overlap, which is noted as a limitation. All literature must have studied caregivers caring for individuals with OC. If the sample included caregivers who were caring for patients with a variety of types of cancer, at least 25% of the sample had to be OC caregivers unless the results explicitly reported on the experience of OC caregivers. Exclusion criteria were: (1) conference abstracts, editorials, opinion pieces, commentaries, and scale and treatment validation studies; (2) studies that do not explicitly report findings for OC caregivers, unless at least 25% of the participants studied are OC caregivers; (3) studies not available in English.

### Step 4: Charting the data

Following study selection, data were charted and synthesized using thematic analysis guided by Braun and Clarke’s [24] Reflexive Thematic Analysis framework. Two reviewers independently analyzed the extracted data, identified recurring patterns, and collaboratively refined overarching themes through discussion and consensus. The 32 studies meeting the inclusion criteria are presented in S1 Table which integrates study characteristics, thematic categories, and corresponding subthemes to provide a comprehensive overview of the literature. S3 Table provides the database-specific search strategies, including search terms, Boolean operators, and subject headings used across databases to enhance transparency and reproducibility. Quantitative data extracted from included studies and referenced in the text are summarized in S2 Table.

### Step 5: Collating, summarizing, and synthesizing the results

Following the guidelines of the PRISMA 2020 flow diagram for new systematic reviews, the titles and abstracts obtained through the search of the five data sets were deduplicated [25]. As is illustrated in Fig 1, 189 duplicates were automatically screened by Covidence and 10 duplicates were identified manually. This resulted in 725 unique titles and articles. An additional 14 articles were identified through manual screening of the reference lists of all articles included in this study, resulting in a final total of 739 unique titles and abstracts. Two reviewers independently screened all titles and abstracts; conflicts were resolved through discussion and consensus. In cases where consensus could not be achieved, a third reviewer was consulted to make the final decision. Upon the completion of the title and abstract screening, 531 articles were excluded as they were deemed irrelevant to this scoping review. As a result, a total of 123 articles were identified for full-text review by two independent reviewers. Of these full text articles, 32 articles met the inclusion criteria. Step six of Arksey & O’Malley’s framework, a consultation exercise with patients, caregivers, or other stakeholders, was not undertaken, as the primary objective of this scoping review was to map and synthesize the existing published literature.

## Results

There has been a substantial increase in research on caregivers of OC patients in recent years. Of the 32 articles included in this scoping review, 27 articles (84.4%) were published in 2010 or later, with the remaining five articles published in 1999, 2002, 2003, 2007, and 2008. Twenty-nine of the 32 articles were based on 29 unique studies, while three articles were derived from one original study in Canada involving 82 caregivers. Of the 30 unique studies, 9 studies were from the USA, 9 from Australia, 8 from Canada, 3 from Turkey, and 1 from Italy. Fifteen of the 30 unique studies used qualitative methodologies, with 13 using quantitative methods and 2 using mixed methods. No articles explored OC caregiving experiences in the low- and middle-income countries (LMIC) which reflects a major gap in this area. A previous scoping review of the literature on caregivers of patients with ovarian cancer was published in 2016, summarizing findings from the relevant articles published prior to November 2014. The findings of the current scoping review largely confirm those of the existing review; however, this review provides updated evidence by also including all relevant articles published after November 2014, offering a more comprehensive and up-to-date understanding of the experiences and needs of OC caregivers.

As shown in S1 and S2 Tables, three main themes were identified.

1. OC caregiver well-being and mental health
2. Challenges facing caregivers and their impact
3. Coping strategies adopted by caregivers

### OC caregiver well-being and mental health

Two sub-themes under the main theme emerged in the literature on OC caregivers’ psychological well-being: (1) Emotional well-being and mental health, discussed in 14 studies, examined caregivers’ experiences of anxiety, depression, and the psychological toll of caregiving; and (2) Grief, anticipatory grief, and bereavement, addressed in 4 studies, explored the emotional challenges caregivers faced following the loss of their loved one, including prolonged grief disorder, PTSD-like symptoms, and unmet bereavement support needs.

#### Emotional wellbeing and mental health.

Many caregivers reported significantly lower mental health compared to population norms [26]. Two major mental health outcomes that emerged were anxiety and depression. Research indicates that the emotional stress experienced by caregivers can sometimes equal or even surpass the stress encountered by their ill spouse, reflecting the immense strain of the caregiving role [27]. Anxiety among caregivers of OC patients was reported as a common concern [28–33]. In Levesque et al., [29] GAD-7 scores indicated mild-to-moderate clinical anxiety, with approximately 42% of male caregivers reporting at least mild symptoms, whereas DiSipio et al. [28] identified anxiety-related unmet needs, such as difficulties coping with uncertainty and managing emotional distress, using the SCNS-P&C44 rather than a clinical measure. Furthermore, many caregivers of OC patients experienced moderate to high levels of anxiety, with 43.9% of one’s study participants having subclinical or clinical levels of anxiety symptoms [31]. Anxiety related to cancer recurrence was noted, although it was not always formally measured [28]. Caregiver anxiety about the potential death of the OC patient was also reported. Caregivers reported significant distress over the anticipated loss, although their anxiety levels remained lower than those of patients [33].

Many caregivers experienced depression [29–31,34,35]. Caregivers reported depression levels which were higher than the reported community norms, with more than half of male caregivers reporting mild (30.6%), moderate (8.3%), or severe (5.6%) levels of depressive symptoms [29]. The most common scale used to measure levels of depression in the quantitative articles was the Hospital Anxiety and Depression Scale (HADS) [31,32,34,35]. One article used the Patient Health Questionnaire-9 (PHQ-9) [29].

The caregiving journey is also marked by considerable emotional hardship, with literature revealing how OC caregivers often struggle to manage their emotions while supporting their partner [36]. Studies show that the psychological wellbeing of family caregivers is significantly shaped by their caregiving role, with patients’ health and emotional challenges directly correlating with caregiver distress [17,37,38]. These emotional challenges for the caregivers are compounded by the growing responsibilities caregivers face as patients lose independence, requiring intensive care [39].

#### Grief, bereavement, adjustment post loved one’s death, and post-traumatic stress disorder (PTSD).

Former caregivers of OC patients often reported having profound grief and bereavement challenges after the death of the OC patients. Bereaved former caregivers frequently experienced shock, trauma, intrusive memories, and symptoms of prolonged grief disorder (PGD) or PTSD-like responses [33,40,41]. Shock and trauma are common experiences among caregivers six months post-loss, highlighting the need for tailored bereavement counseling. However, while earlier research reported that PTSD occurs in 15–40% of bereaved caregivers [41], more recent evidence highlights PGD as a distinct and often more prevalent bereavement-related condition. Reported prevalence rates of PGD vary widely across non-oncology bereavement contexts, including 9.8% in the general population [42], and higher rates following sudden or large-scale loss events, such as COVID-19-related deaths, at 45% [43], and unexpected deaths at 49% [44]. Across the included studies, OC caregivers frequently report PTSD-like symptoms, including anxiety, death anxiety, and severe distress; however, few studies formally assess PTSD or PGD using validated measures, limiting understanding of their prevalence and clinical impact [33,40].

Pre-existing relational dynamics and trauma histories were found to significantly influence grief outcomes, shaping caregiver distress and resilience both during and after the caregiving journey [41]. Unmet needs during the caregiving period—such as emotional support, communication, access to information and resources, and coping strategies—exacerbated post-loss adjustment difficulties, compounding caregivers’ emotional burdens and emphasizing the importance of comprehensive support throughout caregiving, end-of-life, and bereavement to promote better adjustment and well-being [45].

### Challenges facing caregivers and their impact

Seven distinct sub-themes of challenges facing caregivers of OC patients were identified: (1) Escalating needs, disease progression, and patient experiences, discussed in 13 studies, highlighted the impact of increased caregiving demands, worsening patient conditions, and unmet needs on caregivers’ psychological well-being; (2) Interactions with doctors and the medical system, addressed in 9 studies, emphasized caregivers’ struggles with poor communication, lack of access to medical information, and the need for better support from healthcare providers; (3) Social isolation, examined in 10 studies, highlighted the emotional and practical consequences of caregivers’ increasing detachment from family, friends, and community networks; (4) Family responsibilities, discussed in 7 studies, explored the difficulties caregivers faced in balancing caregiving with household duties, childcare, and other family obligations; (5) Work stressors and financial burdens, identified in 6 studies, described the economic strain of caregiving, loss of income, and challenges in maintaining employment while managing caregiving responsibilities; (6) Health behaviours and inadequate self-care, noted in 10 studies, revealed the negative impact of caregiving on caregivers’ physical and mental health, including neglecting their own well-being and adopting unhealthy lifestyle behaviours; and (7) Intrapersonal stressors (e.g., childhood adversities, attachment styles, etc.), examined in 6 studies, highlighted how caregivers’ personal histories, attachment styles, and psychological vulnerabilities influenced their coping capacity and emotional resilience.

#### Escalating needs, disease progression, and patient experiences.

The caregivers’ functional well-being encompasses the ability to perform daily activities, maintain energy levels, fulfill personal and caregiving roles, and attend to self-care [26,28,39], and was shown to decline during the caregiving journey. Decline in functional well-being was particularly prevalent as the disease progression advanced and caregivers struggled to balance responsibilities [26,27,30,31,36,39,45,46]. The caregivers’ unmet needs encompassed access to emotional support for managing caregiving stress, clear and timely information about the patient’s condition and care, and practical assistance with daily tasks, such as managing household responsibilities and coordinating medical care. For example, 56% of caregivers experienced unmet needs 10–12 months before the patient’s death, increasing to 88% in the last three months of the OC patient’s life [26].

Caregivers prioritized reducing the patient’s stress while struggling to balance their own needs, relying on support from family, friends, and healthcare professionals to manage distress during disease recurrence [35]. The emotional burden on caregivers was further exacerbated by the suffering of their loved ones [47]. Distress typically intensified as the patient’s health declined near the end of life, though some families reported a slight reduction in anxiety over time as they adapted to caregiving roles [37]. Additionally, feelings of helplessness in caregiving roles often contributed to depression [40, 41].

#### Interactions with doctors and medical system.

There was considerable variation in the literature assessing the impact on caregivers of interactions with healthcare providers (HCP) including poor communication from HCPs, limited access to patient’s prognostic and treatment information, and HCPs’ insufficient provision of support to address caregiver distress [17,26,30,31,33,47]. For example, some caregivers reported feeling excluded by HCPs and noting a need to rely on self-education to address caregiving responsibilities [17]. This lack of clear communication with the HCP further exacerbated the caregivers’ sense of isolation and stress [17,36]. Caregivers also desired to have more time spent with the doctors to have their questions answered and for information about early indicators of cancer recurrence and methods to reduce risk of recurrence [48]. In contrast, some studies found that poor quality of information and ineffective communication from HCPs did not predict caregiver anxiety and depressive symptoms [30,31,33].

In the final months of the patient’s life, caregivers reported facing intensified challenges, including a heightened need for more tailored information and greater emotional support from HCP, and the wish for more active involvement in medical consultations [26]. Lastly, caregivers also emphasized the importance of better provision of bereavement care within the healthcare system [17].

#### Social isolation.

Social isolation was a significant challenge for many caregivers of OC patients, intensifying with increased caregiving demands and reaching a critical point during the later stages of the disease. During end-of-life care, unmet social needs and heightened distress often led to persistent disconnection from friends and family [17,26,49,50,51]. Despite the growing burden of caregiving as the disease progressed, levels of social support often remained unchanged, leaving caregivers feeling isolated and unsupported as their emotional and practical needs escalated [17,26,36]. Additional resources that address both the physical demands of caregiving tasks and emotional needs have been shown to ease the burden on caregivers, reducing feelings of isolation and improving their overall well-being [39]. However, the ability to manage caregiving demands with adequate practical and emotional resources was closely tied to social support, with OC caregivers who reported higher satisfaction in these systems experiencing improved quality of life and caregiving capacity, while poor social support was linked to greater caregiver distress [26]. Factors contributing to social isolation included the overwhelming nature of caregiving responsibilities, insufficient support from healthcare professionals and peers, limited time for social interactions, and the practice of social distancing from others that some caregivers adopt to manage their stress [45,46]. Prolonged isolation, particularly following the loss of a loved one, was associated with adverse outcomes for caregivers’ well-being, including increased health risks and greater emotional distress [41].

#### Family responsibilities.

OC caregivers’ experiences were strongly influenced by their perceptions of their loved one’s quality of life, and these perceptions shaped how they managed caregiving and family roles [31,49,51]. Balancing caregiving with family obligations often led to heightened stress, particularly when unrelated family stressors compounded burden [27,50]. Declines in a patient’s health often increased their dependency on caregivers, further straining the caregiver’s functional ability to manage household and family obligations [39]. The strain of maintaining household responsibilities intensified during end-of-life care. As unmet needs escalated during this period (see Escalating Needs section), caregivers described inadequate family support and difficulty managing the emotional and practical demands of caregiving, including reducing the patient’s stress and navigating complex family dynamics [26].

On the other hand, family support often plays a critical role in alleviating the emotional burdens of caregiving. Emotional encouragement and practical assistance—such as transportation, meal preparation, and companionship during treatments—were identified as key forms of support that helped distribute caregiving responsibilities and foster a sense of togetherness and unity. This support reduced feelings of isolation among caregivers and created a supportive environment for both patients and caregivers, illustrating the complexity of caregiving dynamics [52].

#### Work stressors and financial burdens.

Financial challenges, including caregiving-related expenses, reduced work hours and difficulty balancing caregiving with professional responsibilities, were identified as a significant contributor to caregiver burden, particularly among lower-income families [15]. Common caregiving-related expenses, including travel, health care supplies, lost productivity, and reduced paid employment hours, often consumed a substantial portion of household income, limiting caregivers’ ability to access proper care and provide adequate support [47]. Financial strain was evident in caregiving households, with spouses identifying significant impacts on their financial stability due to caregiving responsibilities, as measured by the Caregiver Burden Interview [27]. Additionally, caregivers reported economic challenges, including the financial impact of missed work opportunities as they rearranged their lives and priorities to provide full-time care as patients neared the end of life [17]. Furthermore, there is often diminished productivity among caregivers who work outside the home, with absenteeism, presenteeism, and reduced work hours collectively accounting for over a 20% loss in productivity [53]. Balancing caregiving duties and professional responsibilities remained a persistent struggle for many, with unmet financial and work-related needs frequently reported [26,38].

#### Health behaviours and inadequate self-care.

Caregiving responsibilities often significantly impacted caregivers’ health behaviours, as they frequently experienced suboptimal habits, including poorer dietary choices and reduced physical activity, driven by the overwhelming demands of caregiving [17,54]. Negative changes in health behaviours were prevalent among caregivers, with more than half reporting at least one adverse change, such as reduced physical activity or weight gain, while only a small proportion reported improvements like better diet or increased exercise [54]. These adverse changes in health behaviours not only heightened physical health risks but also compounded emotional strain [17,26,49,54].

In addition to these challenges, caregivers were often burdened by the growing physical demands of their role, particularly as patients experienced declining independence and increased need for physical care [36]. This shift frequently left caregivers feeling physically drained and unable to prioritize their own health and well-being [27,40]. However, involving caregivers in the care process, addressing their health needs, and providing practical support have been shown to reduce the severity of physical and emotional strain associated with caregiving responsibilities [28].

Caregivers also frequently prioritized the needs of their loved ones over their own, neglecting their health and well-being [15,26]. Feelings of guilt about taking time for self-care often exacerbated this issue, leading to a persistent imbalance between addressing personal needs and fulfilling caregiving responsibilities [15]. This imbalance not only contributed to poorer health outcomes but also intensified the emotional toll of caregiving [26,47]. Furthermore, the well-being of caregivers was closely tied to patients’ concerns, as some caregivers felt additional stress due to patients’ worries about their welfare, further impacting caregivers’ quality of life [40].

#### Intrapersonal stressors (e.g., childhood adversities, attachment, etc.).

Caregivers of OC patients who were insecurely attached, particularly those with attachment anxiety, were at great risk of caregiver distress, depression, and anxiety [30,31]. Early attachment experiences, such as childhood trauma, which can influence one’s attachment security, appeared to exacerbate emotional stress and caregiving challenges, with insecure attachment styles making caregivers particularly vulnerable to grief and stress under heightened caregiving demands [41,55]. In one study, attachment anxiety accounted for 37% of caregiver distress and accounted for 11% of the anxiety experienced by caregivers [31]. General attachment insecurity yielded mixed results, suggesting variability based on individual circumstances [30].

Men, who represent a significant proportion of those caring for individuals with OC, faced unique challenges. Men often prioritized support with caregiving tasks, such as symptom management, over psychological help for themselves, which highlight the need for gender-specific interventions for caregivers of OC patients [29].

### Caregiver coping strategies

Caregivers of OC patients utilized various strategies to navigate the emotional, psychological, and practical challenges of caregiving, with two primary sub-themes of coping mechanisms emerging: (1) Spirituality, discussed in 6 studies, highlighted the role of prayer, faith, and religious guidance in fostering resilience and emotional stability; and (2) Social support, addressed in 14 studies, emphasized the importance of family, peer networks, and healthcare professionals in mitigating caregiver distress.

#### Spirituality.

Spirituality, through prayer, faith, and guidance from religious figures, served as a crucial coping mechanism for many OC caregivers by fostering resilience, emotional balance, and a sense of meaning amid stress [37,40,47]. Spiritual well-being (SWB) was identified as a consistent protective factor, with higher levels of SWB linked to improved coping and greater emotional resilience, especially when combined with robust social support systems [27]. Spirituality also provided a sense of purpose and optimism, helping caregivers navigate the challenges of their role and sustain their caregiving responsibilities [27,40,56].

A quantitative study, employing the Functional Assessment of Chronic Illness Therapy-Spiritual Well-Being (FACIT-Sp) measurement tool, concluded that caregivers’ spiritual beliefs were strongly associated with their emotional stability across most stages of the cancer trajectory [27]. The universal importance of spirituality was clear, with caregivers of many different faith traditions frequently relying on prayer and religious practices to manage the stress of caregiving and the progression of the disease [47]. However, as the disease advanced and caregiving demands intensified, many caregivers reported a decline in their SWB, finding it increasingly challenging to draw comfort from their spiritual beliefs [17]. Despite this decline, spirituality remained a valuable coping resource for most caregivers, particularly during the earlier stages of the caregiving journey, where it fostered a sense of emotional stability and strength [37].

#### Social support.

Social support was a critical factor in alleviating the psychological and emotional burdens faced by caregivers of OC patients. Studies consistently showed that higher levels of perceived social support were associated with reduced anxiety, depression, hopelessness, and emotional distress [33,35,53]. Social support was measured using the Perceived Social Support Scale, which highlighted its association with reduced emotional distress [33]. The ability to manage caregiving demands with adequate practical and emotional resources was closely tied to social support, with OC caregivers who reported stronger emotional connections to family, friends, and peers experiencing improved quality of life, greater caregiving capacity, and enhanced mental well-being, while poor social support was linked to heightened caregiver distress [26]. Early in the caregiving journey, close relationships provided socioemotional support that enabled caregivers to navigate their responsibilities and roles effectively [37,57]. Peer networks, online forums, and support groups offered opportunities for shared understanding and reduced isolation, particularly during periods of high caregiving stress [47,58].

Despite its importance, many caregivers reported significant unmet social support needs. Time constraints related to caregiving responsibilities often hindered caregivers’ ability to maintain social connections, exacerbating stress and emotional burden [30]. The Cancer Caregiving Tasks, Consequences, and Needs Questionnaire (CaTCoN) was used in one study to identify gaps in caregivers’ access to social networks and connections with other caregivers [34]. Insufficient support from HCPs also remained a challenge, as families were often excluded from psychosocial care programs, which were primarily designed to address patient needs rather than those of caregivers [27,49,55]. These gaps in social support were particularly evident during critical periods, such as the final months of the patient’s life, when 88% of caregivers reported lacking the emotional and practical assistance they needed [26]. Enhancing professional and peer support systems appeared to mitigate caregiver distress, improve quality of life, and reduce feelings of isolation [40,58].

### Identified gaps in the literature

The review process also revealed several substantial gaps in the existing literature. First, the evidence base is geographically limited; all included studies were conducted in high-income countries, with no research emerging from low- or middle-income settings, and with minimal ethno-cultural diversity represented across samples. Second, there was minimal representation of diverse caregiver roles and identities across the literature – including same-sex partners, LGBTQIA2s+ families, siblings, friends, or adult children. Notably, only one study [51] specifically explored the perspectives of children caregivers. Finally, despite consistent reports of unmet informational needs, poor communication with healthcare providers, and significant social isolation, no studies evaluated targeted interventions to improve caregiver support, reduce distress, or enhance family‑centered care. Together, these gaps point to an urgent need for diverse and intervention‑focused research to better understand and support caregivers of individuals living with ovarian cancer.

### Strengths, limitations and implications

The findings from this scoping review offer valuable insights into the multifaceted burden experienced by caregivers of individuals living with ovarian cancer. These findings are consistent with broader literature on cancer caregiving, where similar patterns of emotional distress, role strain, and unmet support needs have been documented across diverse cancer contexts. With respect to emotional distress, studies show that caregivers frequently experience clinically significant levels of anxiety, depression, and psychological burden, often equal to or exceeding that of patients, particularly during advanced disease and end-of-life stages [6]. Regarding role strain, caregivers routinely assume complex and competing responsibilities, including care coordination, symptom management, and household and employment obligations, which contributes to chronic overload, identity disruption, and role conflict [12,13]. Finally, the literature consistently highlights substantial unmet support needs, with caregivers reporting limited access to tailored psychosocial services, clear information from healthcare providers, respite care, and bereavement support, reflecting ongoing gaps in family-centred oncology care systems [12,14]. A key strength of this review is its focus on bringing an up-to-date review of the literature by synthesizing existing literature to highlight the predictors, challenges, coping strategies, and unmet needs faced by caregivers, providing a foundation for addressing gaps in the existing research and practice.

Furthermore, this review provides significant implications of the findings of the current research for clinical practice and policymaking in the field of caregiver experiences and burden in the context of OC, one of the most lethal gynecological cancers. These findings align with economic evidence showing ovarian cancer ranks among the highest in informal caregiving time costs [59]; a recent 11-country analysis estimated USD 471.6 million in annual informal caregiving costs and substantial productivity losses, highlighting the broader societal impact [60]. It also identifies gaps in the literature on caregivers of OC patients and highlights important areas in need of future research.

Notwithstanding these strengths, several limitations of the review must be considered. A limitation of this review is the possible overlap between included reviews and primary studies, which may introduce bias or duplicate data [61]. While an umbrella review can identify such overlaps, our aim was to map the breadth and characteristics of the literature rather than quantifying overlap [62]. The inclusion of non-empirical sources such as peer-reviewed books, narrative reports, and review articles may have introduced overlap or interpretive bias, but these sources were incorporated intentionally to capture the experiential and theoretical perspectives not always present in primary research. However, because reviews may have summarized data from some of the same primary studies we included, there is a potential risk of duplication or overrepresentation of certain findings. This approach reflects the scoping review’s aim to explore the range and nature of available evidence rather than to synthesize outcomes quantitatively.

Additionally, the reviewed studies lack diverse ethno-cultural representation, limiting insights into caregiving experiences across varying cultural, socioeconomic, and geographic contexts. This underrepresentation may restrict the ability to analyze structural inequities and systemic barriers that disproportionately affect certain populations. Furthermore, relevant studies may have been excluded due to the focus on English-language publications. Additionally, the majority of the studies used small, non-representative samples, thereby undermining the generalizability of the reviews’ findings, making it essential to approach conclusions with caution.

While most studies focused on caregivers of patients with advanced or recurrent ovarian cancer, little attention has been given to those supporting individuals living with the disease over longer periods. The caregiving experience in these circumstances may differ significantly, as caregivers navigate ongoing uncertainty and shifting responsibilities across the survivorship trajectory.

Despite these limitations, this scoping review contributes to the understanding of burden of those caring for OC patients and highlights significant areas for future research. Addressing the systemic and structural barriers faced by caregivers should be a priority, particularly for underrepresented populations who may encounter additional challenges in accessing resources and support. Future research should aim to design and evaluate the effectiveness of inclusive, equitable, and culturally sensitive interventions tailored to the diverse needs of caregivers. By prioritizing these areas, stakeholders, including healthcare providers and policymakers, can create more comprehensive caregiver support systems, with the aim to improve both caregiver well-being and patient outcomes.

## Conclusion

The caregiving experience is marked by significant complexity, so it demands a nuanced understanding of the various roles and challenges they face, and the evolving needs of the OC patients for whom they are caring. This scoping review highlights the substantial burdens caregivers face, encompassing emotional, physical, and financial domains. Key findings revealed that OC caregivers are at high risk of anxiety, depression, and social isolation, problems which are compounded by the unpredictable progression of the disease and its high recurrence rates [8,16]. While coping mechanisms such as spirituality and social support proved very helpful for the well-being of many caregivers, they were often insufficient to address the excessive demands on caregivers [27,37].

This review highlights an urgent need for tailored mental health, respite care, and targeted support interventions for caregivers of OC patients. However, there remains a lack of research from LMICs, as well as limited evidence on the development and evaluation of targeted interventions; studies also rarely include ethno-culturally diverse populations or male caregivers’ unique experiences. By addressing these disparities and integrating caregiver perspectives into healthcare policies and practices, future research and program development can play an instrumental role in improving both caregiver and patient outcomes. Transforming the caregiving experience of those who provide care to individuals with OC requires a concerted effort to bridge these gaps, ensuring that caregivers receive the support needed to navigate their roles effectively.

## Supporting information

S1 Table Studies included in the scoping review. Author, year, country, study design, objectives, sample characteristics, and key themes.
(DOCX)

S2 Table Extracted quantitative findings and measurement tools. Summary of reported quantitative outcomes and instruments used.
(DOCX)

S3 Table Database-specific search strategies. Full electronic search strategies, including search terms, Boolean operators, and subject headings.
(DOCX)

S4 Table Thematic coding framework. Coding structure and synthesis of qualitative themes and subthemes.
(DOCX)
