# Supplementary material for: Exploring the experiences, challenges, and coping strategies of caregivers of women with Ovarian Cancer: A scoping review
Source: PLoS One. 2026 Apr 30;21(4):e0345325. doi: 10.1371/journal.pone.0345325 (PMC13132214; doi:10.1371/journal.pone.0345325)
Supplement: S1 Table — Author, year, country, study design, objectives, sample characteristics, and key themes. (DOCX) [file pone.0345325.s001.docx]

**Supplementary Table 1: Studies Included in the Scoping Review by Author, Year of Publication, Study Design, Study Objectives, Study Sample and Themes**

| **Author(s) (year)** | **Country** | **Study Design** | **Study Objectives** | **Study Sample** | **Key Themes Identified** |
| --- | --- | --- | --- | --- | --- |
| Angioli et al. (2015) | Italy | Cross-sectional survey | To investigate the economic changes experienced by family caregivers of advanced OC during first-line treatment. | Family caregivers of individuals living with advanced OC (n = 172) | Work stressors and financial burdens; Social support |
| Beesley et al. (2011) | Australia | Quantitative study | To explore behavioural changes in OC caregivers and the factors influencing them. | Caregivers of individuals living with OC (n = 101) | Neglecting health and self-care |
| Butow et al. (2014) | Australia | Prospective cohort study | To explore quality of life and unmet needs of caregivers of women living with OC. | Caregivers of women with newly diagnosed epithelial OC (n = 99) | Quality of life and emotional wellbeing; Escalating needs and disease progression; Interactions with the medical system; Social isolation; Family responsibilities; Work stressors and financial burdens; Neglecting self-care; Social support |
| DiSipio et al. (2024) | Australia | Prospective cohort survey | To explore the care needs of individuals with OC and their caregivers at various points between the initial diagnosis and recurrence. | Individuals with OC (n = 288), OC caregivers (n = 140) | Quality of life and emotional wellbeing; Anxiety and depression; Escalating needs and disease progression |
| De Rooijet al. (2018) | USA | Qualitative study | To describe the challenges experienced by individuals with OC, their caregivers, and care providers following treatment, and their survivorship care preferences. | Individuals with OC (n = 13), OC caregivers (n = 9), healthcare providers (n = 8) | Interactions with the medical system |
| Ferrell et al. (2002) | USA | Content analysis | To explore the quality of life of OC family caregivers. | OC caregivers (n = 1,100 correspondences) | Quality of life and emotional wellbeing; Grief and bereavement; Escalating needs and disease progression; Spirituality; Social support |
| Frost et al. (2012) | USA | Longitudinal qualitative study | To compare the spiritual wellbeing and quality of life of women diagnosed with OC and their spouses. | Individuals with OC (n = 70), spouses (n = 26) | Quality of life and emotional wellbeing; Escalating needs and disease progression; Family responsibilities; Spirituality; Social support |
| Güler et al. (2020) | Turkey | Qualitative study | To explore what family members of individuals with OC shared online between 2009 and 2019. | Online posts by family members (n = 78) | Interactions with the medical system; Work stressors and financial burdens; Neglecting health and self-care; Spirituality; Social support |
| Hand et al. (2019) | USA | Delphi approach | To explore the unmet and support needs of caregivers of individuals with gynaecologic cancer. | Caregivers (n = 16), clinicians and advocates (n = 16) | Social support |
| Hartnett et al. (2016) | USA | Cross-sectional survey | To examine the level of burden experienced by caregivers and the factors associated with caregiver burden among individuals with end-stage OC. | Caregivers of individuals with end-stage OC (n = 50) | Work stressors and financial burdens; Neglecting health and self-care |
| Jayde & Boughton (2016) | Australia | Qualitative study | To explore and interpret the lived experience of maternal OC for adult children prior to recurrence. | Adult children of women diagnosed with OC (n = 9) | Emotional wellbeing; Anxiety and depression; Social isolation |
| Koldjeskiet al. (2007) | USA | Mixed-method study | To examine the impact of caregiving responsibilities on family functioning among family caregivers of individuals with OC. | Families of individuals with OC (n = 18) | Quality of life and emotional wellbeing; Escalating needs and disease progression; Spirituality; Social support |
| Levesque et al. (2022) | Australia | Cross-sectional survey | To explore the challenges faced by male caregivers of individuals with OC and their psychosocial wellbeing. | Male caregivers of individuals with OC (n = 36) | Anxiety and depression; Intrapersonal stressors (e.g., attachment, trauma) |
| Le et al. (2003) | Canada | Prospective cohort study | To assess quality of life among caregivers of women with OC receiving chemotherapy and examine correlations with patients’ quality of life scores. | Patient-caregiver pairs (n = 30) | Quality of life and emotional wellbeing; Escalating needs and disease progression |
| McLean & Hales(2010) | Canada | Case study | To describe the psychosocial distress and risks of patients with end-stage cancer and their caregivers, particularly with challenging attachment stylesor trauma histories. | Patient with metastatic OC (n = 1) and spouse (n = 1) | Intrapersonal stressors; Social support |
| Petricone-Westwood et al. (2021a) | Canada | Cross-sectional survey | To examine how attachment insecurity and experiences with cancer care influence depression, anxiety, and caregiving experiences. | Partners of patients with OC (n = 82) | Anxiety and depression; Escalating needs and disease progression; Interactions with the medical system; Intrapersonal stressors; Social support |
| Petricone-Westwood et al. (2021b) | Canada | Post-hoc analysis | To investigate associations between distress and caregiving experiences within the healthcare system. | Partners of patients with OC (n = 82) | Anxiety and depression; Interactions with the medical system; Intrapersonal stressors |
| Petricone-Westwood et al. (2022) | Canada | Post-hoc analysis | To assess whether attachment styles and caregiving experiences predict fear of cancer recurrence. | Partners of patients with OC (n = 82) | Anxiety and depression; Social support |
| Petricone-Westwood & Lebel 2016) | Canada | Scoping Review | To explore the existing literature on caregivers of individuals living with OC. | 19 articles (9 quantitative, 5 qualitative, 2 mixed-methods, 2 case studies, 1 personal account) | **Quality of life and emotional wellbeing; Interactions with the medical system; Social isolation; Neglecting health and self-care; Spirituality** |
| Price et al. (2010) | Australia | Prospective cohort study | To evaluate depression and anxiety among OC patients and caregivers compared to community norms. | Individuals with OC (n = 798), caregivers (n = 373) | Anxiety and depression; Escalating needs and disease progression; Social support |
| Sanderson et al. (2013) | Australia | Mixed-methods study | To identify post-traumatic stress symptoms among bereaved caregivers of OC patients. | Bereaved caregivers of individuals with OC (n = 32) | Grief and bereavement; Escalating needs and disease progression; Social isolation; Intrapersonal stressors |
| Stilos et al. (2018) | Canada | Qualitative study | To investigate experiences of family caregivers providing care for loved ones with advanced OC. | Family caregivers of individuals with OC (n = 13) | Interactions with the medical system; Social isolation; Family responsibilities; Neglecting health and self-care; Social support |
| Stragapede et al. (2023) | Canada | Cross-sectional survey | To explore the relationships between OC caregivers’ perceptions of patient quality of life and their own caregiving impacts. | Spousal caregivers of individuals with OC (n = 82) | Quality of life and emotional wellbeing; Escalating needs and disease progression; Interactions with the medical system; Social isolation; Family responsibilities |
| Tan et al. (2020) | Australia | Qualitative study | To explore subjective experiences of individuals with OC and their caregivers navigating uncertainty. | Individuals with OC (n = 219), caregivers (n = 78) | Escalating needs and disease progression; Social isolation |
| Uslu-Sahan, Terzioglu & Koc (2019) | Turkey | Cross-sectional survey | To assess differences in hopelessness and death anxiety between hospitalized patients with gynaecologic cancers and their caregivers. | Patients (n = 200), caregivers (n = 200) | Anxiety and depression; Grief and bereavement; Social support |
| Webb et al. (2022) | Australia | Qualitative study | To explore fear of cancer recurrence among caregivers of individuals living with OC. | Caregivers of individuals with OC (n = 24) | Escalating needs and disease progression; Social isolation |
| Wice (2019) | USA | Narrative | To describe the experience of OC from a caregiver’s perspective who is a healthcare provider. | Not applicable | Social support |
| Teskereci& Kulakac(2016) | Turkey | Mixed-methodssystematic review | To explore life experiences of family caregivers supporting women with gynaecologic cancer. | 16 articles (10 quantitative, 5 qualitative, 1 mixed) | Social isolation |
| Tarraza & Ellerkmann (1999) | USA | Qualitative study | To explore the impact of OC on family members. | Families of individuals with OC (n = 32) | Quality of life and emotional wellbeing; Anxiety and depression; Escalating needs and disease progression |
| Ponto & Barton (2008) | USA | Qualitative study | To describe OC from the perspective of spouses of affected individuals. | Spouses of individuals with OC (n = 11) | Family responsibilities |
| Yaşar & Terzioğlu(2022) | Turkey | Descriptive quantitative study | To evaluate the caregiving burden and quality of life among caregivers of patients with gynaecologic cancer and identify sociodemographic influences. | Caregivers of women with ovarian cancer (n = 118) | Quality of life and emotional wellbeing; Anxiety and depression; Work stressors and financial burdens; Neglecting health and self-care; Social support |
| Vardar & Serçekuş(2023) | Turkey | Qualitative study | To explore experiences of Muslim women with gynaecologic cancers and their family caregivers, including difficulties, coping, and expectations of health services. | Family caregivers of women with ovarian cancer (n = 8) | Emotional wellbeing; Anxiety and depression; Escalating needs and disease progression; Interactions with the medical system; Social isolation; Family responsibilities; Work stressors and financial burdens; Spirituality; Social support |
