# Supplementary material for: Exploring the experiences, challenges, and coping strategies of caregivers of women with Ovarian Cancer: A scoping review
Source: PLoS One. 2026 Apr 30;21(4):e0345325. doi: 10.1371/journal.pone.0345325 (PMC13132214; doi:10.1371/journal.pone.0345325)
Supplement: S2 Table — Summary of reported quantitative outcomes and instruments used. (DOCX) [file pone.0345325.s002.docx]

**Supplementary Table B: Data Extraction Table of Studies Included in the Scoping Review by Author, Year of Publication, Study Design, Study Objectives, Study Sample, Key Themes, Quantitative Findings, Measurement Tools**

| **Author(s) (year)** | **Country** | **Study Design** | **Study Objectives** | **Study Sample** | **Key ThemesIdentified** | **Quantitative FindingsReported in Manuscript Text** | **Measurement Tool / Scale Used** |
| --- | --- | --- | --- | --- | --- | --- | --- |
| **Angioli et al. (2015)** | Italy | Cross-sectional survey | To investigate economic changesexperienced by family caregivers during first-line treatment. | Caregivers of advanced OC (n = 172) | Work stressors; Financial burdens; Social support | >20% loss in productivity | Caregiver Burden Interview / productivity metrics |
| **Beesleyet al. (2011)** | Australia | Quantitative study | To explore behaviouralchangesincaregivers and influencingfactors. | Caregivers of OC (n = 101) | Neglecting health and self-care | >50% reported ≥1 adverse health-behaviourchange | Custom behaviourquestionnaire |
| **Butow et al. (2014)** | Australia | Prospective cohort study | To explore quality of life and unmet needsamongcaregivers. | Caregivers of newly diagnosed epithelial OC (n = 99) | QoL; Emotional wellbeing; Escalating needs; Medical interactions; Social isolation; Family responsibilities; Work/financial burdens; Self-care; Social support | 56% unmet needs(10–12 monthspre-death); 88% unmet needs(last 3 months) | SCNS-P&C44 |
| **DiSipio et al. (2024)** | Australia | Prospective cohort survey | To explore careneeds of OC patientsandcaregivers from diagnosistorecurrence. | Patients(n = 288); Caregivers (n = 140) | QoL/emotional wellbeing; Anxiety & depression; Escalating needs | N/A | N/A |
| **De Rooij et al. (2018)** | USA | Qualitative study | To describe challenges post-treatment and survivorship preferences. | Patients(n = 13); Caregivers (n = 9); HCPs (n = 8) | Medical system interactions | N/A | N/A |
| **Ferrell et al. (2002)** | USA | Content analysis | To explore quality of life of OC family caregivers. | OC caregivers (n = 1,100 letters) | QoL; Grief/bereavement; Escalating needs; Spirituality; Social support | N/A | N/A |
| **Frost et al. (2012)** | USA | Longitudinal qualitative | To compare spiritual wellbeing and QoL of OC patientsand spouses. | Patients(n = 70); Spouses(n = 26) | QoL; Escalating needs; Family responsibilities; Spirituality; Social support | N/A | N/A |
| **Güler et al. (2020)** | Turkey | Qualitative study | To explore online posts by caregivers. | Family member posts (n = 78) | Medical system interactions; Work/financial stress; Neglecting self-care; Spirituality; Social support | N/A | N/A |
| **Hand et al. (2019)** | USA | Delphi study | To explore unmet needs of gynecologic cancer caregivers. | Caregivers (n = 16); Clinicians (n = 16) | Social support | N/A | N/A |
| **Hartnett et al. (2016)** | USA | Cross-sectional survey | To examine caregiver burden and associated factors. | Caregivers of end-stage OC (n = 50) | Work/financial burdens; Neglecting self-care | N/A | N/A |
| **Jayde & Boughton (2016)** | Australia | Qualitative study | To explore lived experience of maternal OC in adult children. | Adult children (n = 9) | Emotional wellbeing; Anxiety/depression; Social isolation | N/A | N/A |
| **Koldjeski et al. (2007)** | USA | Mixed-methods | To examine caregiving impact on family functioning. | Families(n = 18) | QoL; Escalating needs; Spirituality; Social support | N/A | N/A |
| **Levesque et al. (2022)** | Australia | Cross-sectional survey | To explore challenges among male caregivers. | Male caregivers (n = 36) | Anxiety/depression; Intrapersonal stressors | 42% ≥ mild anxiety; 30.6% mild, 8.3% moderate, 5.6% severe depression | GAD-7; PHQ-9 |
| **Le et al. (2003)** | Canada | Prospective cohort study | To assesscaregiverQoL during chemotherapy. | Patient-caregiver pairs(n = 30) | QoL; Escalating needs | N/A | N/A |
| **McLean & Hales(2010)** | Canada | Case study | To describe psychosocial distresswithtrauma/attachment issues. | Patient (n = 1), Spouse (n = 1) | Intrapersonal stressors; Social support | N/A | N/A |
| **Petricone-Westwood et al. (2021a)** | Canada | Cross-sectional survey | To examine attachmentinsecurity, cancer care experiences, and distress. | Partners(n = 82) | Anxiety/depression; Escalating needs; Medical system interactions; Intrapersonal stressors; Social support | N/A | N/A |
| **Petricone-Westwood et al. (2021b)** | Canada | Post-hoc analysis | To investigate distressandcaregiving experiences. | Partners(n = 82) | Anxiety/depression; Medical interactions; Intrapersonal stressors | 43.9% subclinical/clinical anxiety | HADS-A |
| **Petricone-Westwood et al. (2022)** | Canada | Post-hoc analysis | To assesswhetherattachment and caregiving experiences predict fear of recurrence. | Partners(n = 82) | Anxiety/depression; Social support | N/A | N/A |
| **Petricone-Westwood & Lebel (2016)** | Canada | Scoping review | To explore existing OC caregiver literature. | 19 included papers | QoL; Medical interactions; Social isolation; Neglecting self-care; Spirituality | N/A | N/A |
| **Price et al. (2010)** | Australia | Prospective cohort | To evaluate depression/anxiety vscommunitynorms. | Patients(n = 798); Caregivers (n = 373) | Anxiety/depression; Escalating needs; Social support | N/A | N/A |
| **Sanderson et al. (2013)** | Australia | Mixed-methods | To identifyPTSD symptoms in bereaved caregivers. | Bereaved caregivers (n = 32) | Grief/bereavement; Escalating needs; Social isolation; Intrapersonal stressors | PTSD prevalence reported: 15–40% | Various PTSD measures (not specified) |
| **Stilos et al. (2018)** | Canada | Qualitative | To investigate experiences of family caregivers of advanced OC. | Caregivers (n = 13) | Medical interactions; Social isolation; Family responsibilities; Neglecting self-care; Social support | N/A | N/A |
| **Stragapede et al. (2023)** | Canada | Cross-sectional survey | To examine caregiver QoL and caregiving impacts. | Spousal caregivers (n = 82) | QoL; Escalating needs; Medical interactions; Social isolation; Family responsibilities | N/A | N/A |
| **Tan et al. (2020)** | Australia | Qualitative | To explore experiencesnavigatinguncertainty. | Patients(n = 219); Caregivers (n = 78) | Escalating needs; Social isolation | N/A | N/A |
| **Uslu-Sahan et al. (2019)** | Turkey | Cross-sectional survey | To assesshopelessness and death anxiety in gynecologic cancer caregivers. | Patients(n = 200); Caregivers (n = 200) | Anxiety/depression; Grief/bereavement; Social support | N/A | N/A |
| **Webb et al. (2022)** | Australia | Qualitative study | To explore fear of cancer recurrence in caregivers. | Caregivers (n = 24) | Escalating needs; Social isolation | N/A | N/A |
| **Wice (2019)** | USA | Narrative | To describe OC experience from a caregiver-clinician. | N/A | Social support | N/A | N/A |
| **Teskereci & Kulakac(2016)** | Turkey | Mixed-methods review | To explore life experiences of caregivers of gynecologic cancer patients. | 16 studies | Social isolation | N/A | N/A |
| **Tarraza& Ellerkmann(1999)** | USA | Qualitative study | To explore OC’s impact on family members. | Family members (n = 32) | QoL; Anxiety/depression; Escalating needs | N/A | N/A |
| **Ponto & Barton (2008)** | USA | Qualitative study | To describe OC from spouses’ perspectives. | Spouses(n = 11) | Family responsibilities | N/A | N/A |
| **Yaşar & Terzioğlu(2022)** | Turkey | Descriptive quantitative | To evaluate caregiving burden and QoL. | Caregivers of women with OC (n = 118) | QoL; Anxiety/depression; Work/financial burdens; Self-care; Social support | N/A | N/A |
| **Vardar & Serçekuş (2023)** | Turkey | Qualitative study | To explore experiences of Muslim women with gynecologic cancersand their caregivers. | Caregivers (n = 8) | Emotional wellbeing; Anxiety/depression; Escalating needs; Medical interactions; Social isolation; Family responsibilities; Work/financial burdens; Spirituality; Social support | N/A | N/A |
