# Supplementary material for: Exploring the experiences, challenges, and coping strategies of caregivers of women with Ovarian Cancer: A scoping review
Source: PLoS One. 2026 Apr 30;21(4):e0345325. doi: 10.1371/journal.pone.0345325 (PMC13132214; doi:10.1371/journal.pone.0345325)
Supplement: S3 Table — Full electronic search strategies, including search terms, Boolean operators, and subject headings. (DOCX) [file pone.0345325.s003.docx]

**Supplementary Table C: Search Terms, Date of Search and Databases Searched**

| Text Words  (used in all databases) |  | cancer* or sarcoma* or tumo* or malignan* or carcino* or adenocarcinoma* or neoplasm*    ova* or ovum* or germinal* or gonad* or epithel* or granulosa* or germ cell* or stromal*    caregiv* or carer* or caretak* or care partner*    Luteoma or Meigs Syndrome or Sertoli-Leydig or Thecoma* or Brenner* or Germinal epithelium or dysgerminoma or serous carcinoma | | |
| --- | --- | --- | --- | --- |
| Time and Date of Search | Database | Subject Headings Specific to Database | Search Syntax (Reconstructed) | Results |
| October 22, 2024, 3 PM | Medline (Ovid) | Neoplasms  Ovary  Ovarian neoplasms  Caregivers  Caregiver burden | (exp Neoplasms/ OR cancer*.ti,ab. OR sarcoma*.ti,ab. OR tumo*.ti,ab. OR malignan*.ti,ab. OR carcino*.ti,ab. OR adenocarcinoma*.ti,ab. OR neoplasm*.ti,ab.) AND (exp Ovarian Neoplasms/ OR ova*.ti,ab. OR ovum*.ti,ab. OR germinal*.ti,ab. OR gonad*.ti,ab. OR epithel*.ti,ab. OR granulosa*.ti,ab. OR "germ cell*".ti,ab. OR stromal*.ti,ab. OR Luteoma.ti,ab. OR "MeigsSyndrome".ti,ab. OR "Sertoli-Leydig".ti,ab. OR Thecoma*.ti,ab. OR Brenner*.ti,ab.) AND (exp Caregivers/ OR caregiv*.ti,ab. OR carer*.ti,ab. OR caretak*.ti,ab. OR "care partner*".ti,ab. OR "caregiver burden".ti,ab.) LIMIT TO (English language AND humans) | 164 |
| October 22, 2024, 3 PM | PsychINFO (Ovid) | Cancers  Ovaries  Caregiver Burden  Caregivers  Family Caregivers  Caregiver Attitudes | (DE "Cancer" OR cancer*.ti,ab. OR neoplasm*.ti,ab. OR carcino*.ti,ab. OR tumo*.ti,ab.) AND (DE "Ovaries" OR ovarian.ti,ab. OR ova*.ti,ab. OR gonad*.ti,ab. OR epithel*.ti,ab.) AND (DE "Caregivers" OR DE "Caregiver Burden" OR DE "Family Caregivers" OR caregiv*.ti,ab. OR carer*.ti,ab. OR caretak*.ti,ab. OR "care partner*".ti,ab.) LIMIT TO (peer-reviewed journal AND English language) | 33 |
| October 22, 2024, 3 PM | Embase (Ovid) | Neoplasm  Ovary cancer  Ovary  Caregiver Caregiver support  Caregiver Strain Index  Caregiver burden  Caregiver burnout  Caregiver quality of life index-cancer | ('ovarian cancer'/exp OR 'ovarian carcinoma':ti,abOR'epithelial ovarian tumor':ti,abOR'germ cell ovarian cancer':ti,ab OR 'granulosa cell tumor':ti,ab OR 'stromal tumor':ti,ab) AND ('caregiver'/exp OR caregiv*:ti,ab OR carer*:ti,ab OR "care partner*":ti,ab OR 'caregiver burden'/exp OR 'caregiver burnout':ti,ab OR 'caregiver strain':ti,ab) AND ('psychological stress'/exp OR 'quality of life'/exp OR 'emotional burden':ti,ab) LIMIT TO (humans AND English language) | 441 |
| October 22, 2024, 3 PM | Social Work Abstracts (Ovid) | *Database search does not utilize subject headings. Only identified keywords were used. | N/A | 0 |
|  | CINAHL (EBSCO) | Neoplasms  Cancer patients  Cancer pain  Chemotherapy, cancer  Rehabilitation, cancer  Cancer survivors  Carcinoma  Cancer fatigue  Neoplasm metastasis  Neoplasms, second primary  Neoplastic syndromeshereditary  Oncologic care  Neoplasms, radiation-induced  Oncology surgery  Psycho-ocology  Oncology care units  Ovary  Ovum  Ovarian follicle  Gonads  Carcinoma, Ovarian Epithelial  Ovarian Neoplasms  Neoplasms, Germ Cell and Embryonal  Granulosa Cell Tumor  Genes, BRCA  Neoplasms, Gonadal Tissue  Hereditary Breast and Ovarian Cancer Syndrome  Caregiver burden  Caregiver attitudes  Caregiver support  Caregivers | (MH "Ovarian Neoplasms+" OR "ovarian cancer*" OR "epithelial ovarian carcinoma*" OR "ovarian tumor*" OR "ovarian malignancy*") AND (MH "Caregivers+" OR "Caregiver Burden" OR "Family Caregivers" OR caregiv* OR carer* OR "care partner*" OR "caregiver stress") AND (MH "Psychosocial Factors+" OR "Quality of Life" OR "Emotional Adjustment" OR burden OR stress) Limiters: English Language; Peer-Reviewed; Publication Year 2000–2024 | 87 |
| TOTAL: |  |  |  | 725 |
